# Aversive Event Anticipation Affects Connectivity between the Ventral Striatum and the Orbitofrontal Cortex in an fMRI Avoidance Task

**DOI:** 10.1371/journal.pone.0068494

**Published:** 2013-06-24

**Authors:** Ingeborg Bolstad, Ole A. Andreassen, Greg E. Reckless, Niels P. Sigvartsen, Andres Server, Jimmy Jensen

**Affiliations:** 1 KG Jebsen Centre for Psychosis Research, Division of Mental Health and Addiction, Oslo University Hospital, Oslo, Norway; 2 Institute of Clinical Medicine, University of Oslo, Oslo, Norway; 3 Department of Neuroradiology, Oslo University Hospital, Oslo, Norway; 4 Centre for Psychology, Kristianstad University, Kristianstad, Sweden; Bellvitge Biomedical Research Institute-IDIBELL, Spain

## Abstract

Ability to anticipate aversive events is important for avoiding dangerous or unpleasant situations. The motivation to avoid an event is influenced by the incentive salience of an event-predicting cue. In an avoidance fMRI task we used tone intensities to manipulate salience in order to study the involvement of the orbitofrontal cortex in processing of incentive salience. In the task, cues predicting either aversive or neutral avoidable tones were presented. Ventral striatum, amygdala and anterior insula activations were significantly stronger during presentation of cues for aversive than neutral tones. A psychophysiological interaction analysis showed stronger connectivity between the ventral striatum and the orbitofrontal cortex during aversive than neutral conditions. The present study shows an interaction between the ventral striatum, a structure previously linked to negative incentive salience, and the orbitofrontal cortex supporting a role for this region in processing salience. In addition, this study replicates previous findings suggesting that the task is robust.

## Introduction

Being able to look ahead of the present moment and anticipate upcoming events gives major benefits. For example, by anticipating a future negative event we can adjust behavior and prepare actions that might enable us to avoid an unpleasant or dangerous situation. The anticipation of future events depends on utilizing cues in our environment. When a neutral cue becomes associated with a positive or negative event, incentive salience is assigned and motivates behavior to approach or avoid situations, respectively [1,2]. Incentive salience has been defined as a type of motivation that promotes approach towards a reward [3,4], and subsequent research has shown that this concept also includes avoidance from punishment [1]. Reward processing and its neural underpinnings have been quite extensively explored. Whether the processing of its aversive counterpart is similar and which brain regions are involved is a less investigated area of research and also disputed. The present study investigated anticipation specifically during aversive conditions.

Salience constitutes the motivational value of a stimulus but not its hedonic impact [4]. Anticipating future adversity and initiation of goal-directed behavior, as for example when attempting to avoid an unpleasant event, involves several cognitive and affective processes in which motivation plays a fundamental role. While several different brain regions have been shown to be involved in incentive salience, the connectivity between these regions has not been fully explored. Incentive salience is primarily mediated by mesolimbic pathways [3,4]. The mesolimbic system has traditionally been conceptualized as a reward system but a growing literature suggests that it may rather serve a function in processing incentive salience. This is supported by studies showing that not only appetitive, but also aversive events are processed by this system [5–7]. When a cue-outcome association is acquired, significance shifts from the outcome to the cue. The cue that was originally neutral takes on motivational properties that guide behavior towards or away from the outcome [4,8]. Accordingly, several studies have shown that the ventral striatum (VS) responds more during the anticipation stage than at the outcome stage for both appetitive and aversive events [8,9]. Thus, the concepts of anticipation and incentive salience are closely related, and this is mirrored in the underpinning neurobiology [2,10,11].

In addition to the VS, the anterior insula and the anterior cingulate cortex (ACC) has repeatedly been implicated in salience processing [12,13], anticipation related processes [14–16] and fear conditioning [17]. These regions seem to serve a function in motivational control of the behavior as communicating critical information between the amygdala, the VS and sensory and motor areas. Further, a stimulus that forecasts an opportunity to avoid an aversive event is highly relevant to the individual and recent reports indicate that the amygdala is important in this context [18]. Studies have shown that the amygdala responds to motivationally relevant stimuli rather than the emotional content [19], and that the amygdala response is related to stimulus intensity [20]. This research seems to point to a role for amygdala in an early stage of the anticipation process, before a stimulus necessarily gains incentive salience, serving a function in relevance detection [19,21] and allocating personal value to the stimulus.

Anticipation involves decoding stimulus value and valence, assessing the importance of the upcoming event and using this information to guide behavior. The orbitofrontal cortex (OFC) has been implicated in these processes, and is considered to be important for motivation. The OFC is well positioned to play a part in this process as it is reciprocally connected to other important motivation areas such as the VS, amygdala and hippocampus, and it also receives multimodal sensory information [22]. Many studies implicate the OFC in motivation, however most studies have used appetitive stimuli and a majority has looked at how the OFC behaves during the outcome phase of prediction, and how the interaction between the VS and OFC is affected by differential incentive salience has not yet been explored.

The primary aim of this study was to explore functional connectivity between the VS and the OFC comparing anticipation of avoidable negative salient events and neutral events. Because the VS is thought to mediate incentive salience and the OFC is proposed to be involved in coding of motivational significance of the anticipated event and guide behavior based on this, it was hypothesized that the VS and the OFC would be more functionally connected during aversive as compared to neutral conditions. In addition, as the OFC and the amygdala have reciprocal anatomical connections and are thought to play roles in anticipation, motivation and relevance detection an increase in functional connectivity between these regions during aversive conditions was hypothesized.

In previous studies that have targeted brain activation during incentive salience, a range of stimulus modalities have been employed, such as monetary loss, aversive pictures, taste, smell or sound. Jensen et al. [5] studied incentive salience by employing an instructed avoidance task where two different cues predicted an aversive electric shock or a neutral event, respectively. In the present study our secondary aim was to replicate these findings by testing whether a similar paradigm, but with auditory outcome stimuli, would yield activations in the same brain areas. Thus, we expected activations in these regions of interest: the VS because of its central role in motivational salience; the amygdala because it is implicated in personal relevance detection; the anterior insula and the ACC because these regions are involved in monitoring and integrating relevant information. To examine these issues an fMRI task based on avoidance was employed, and a psychophysiological interaction analysis (PPI) was performed to address the hypotheses regarding functional connectivity.

## Methods

### *Participants*

This study was approved by the Norwegian Regional Committees for Medical and Health Research Ethics. Nineteen subjects were included in the study after giving written informed consent and passing a health screening. None of the subjects were taking medication. The participants had responded to posted advertisement. Three data sets were excluded from analysis because of excessive movement during scanning. Sixteen subjects (eight females; 26±6 years of age) were included in the analysis. The participants were financially compensated after completing their participation.

### *Task*

We employed an event-related fMRI paradigm based on avoidance. During one trial of the task, the subjects would first see either a turquoise or an orange circle against a black background, for two seconds. The circle preceded a target (a black screen with a yellow star) at which the subjects were instructed to respond by pushing a button with the right index finger as quickly as possible in order to avoid a subsequent sound. One colored circle predicted an aversive sound (fire truck hoot) and the other colored circle predicted a low-volume non-aversive tone. Before the task started the aversive sound was individually titrated. This was done while the subject was in the scanner and an fMRI scanner sequence was running in order to have relevant background noise. The subjects used button presses to adjust the level of sound intensity. When the sound reached the subjective level “unpleasant, but tolerable”, the subjects indicated this by pressing another button, and the indicated sound level was used in task that followed after the sound titration procedure. During the task the subjects were instructed to act on every target and told that if they were able to press sufficiently quickly they could avoid the sound (both in aversive and non-aversive trials). After they had responded, there was either 1.5 s of sound or silence depending on the how fast they were able to respond. The task contained 80 trials (40 aversive and 40 non-aversive) and was designed to adapt to individual response times so that a sound would be presented in approximately 20% of the trials. The subjects were told beforehand which color predicted which sound, and that an aversive or a non-aversive sound would be heard in some trials depending on their response quickness, but they were not informed about the outcome schedule. The colors were counterbalanced and the trial sequence was randomized across subjects. The time interval between cue onset and consequence onset was on average 4.7±0.8 s, and the inter-trial-interval was jittered between 3–7 s with an average duration of 5 s.

### *fMRI data acquisition*

#### Apparatus

The E-Prime software (Psychology software tools Inc., Pittsburgh, Pennsylvania, USA) was used to program the task and to control the experiment. In the scanner the stimuli were presented through VisualSystem goggles (Nordic Imaging Lab, Bergen, Norway, http://www.nordicneurolab.com) and responses collected by ResponseGrip (Nordic imaging Lab, Bergen, Norway, http://www.nordicneurolab.com). Sounds were delivered through headphones (Nordic Imaging Lab, Bergen, Norway, http://www.nordicneurolab.com).

#### Image acquisition

The examinations were performed on a 3T General Electric, Signa HDxt scanner (GE Healthcare, Milwaukee, WI, USA). The MR imaging protocol consisted of a T2*-weighted sequence in the transverse plane with the following parameters: repetition time (TR) = 2000 ms; echo time (TE) = 25 ms and flip angle = 78 degrees. One run of 454 (±3, depending on individual response times) volumes was collected for each individual (36 slices of 3.5 mm thickness, 0.5 mm gap, sequential acquisition, in plane resolution 4 x 4 mm, 64 x 64 matrix). Three dummy disc acquisitions were acquired initially during each scan and discarded. Structural T1-weighted images (TR = 10.9 s; TE = 4.6 s; flip angle = 13 deg; 236 axial slices of 0,6 mm thickness; 0,5 mm x 0,5 mm in-plane resolution, 512 x 512 matrix) were obtained prior to the functional series and submitted to radiological screening to identify subjects with possible anatomical abnormalities.

### *Image analysis*

#### Pre-processing

DICOM image files were converted to NIfTI-1 format through the NordicICE software (Nordic Imaging Lab, Bergen, Norway, http://www.nordicneurolab.com). Raw data were visually inspected to ensure image quality. Three subjects data were excluded securing no subjects included in the analysis moved more than 3 mm in any direction through the experiment. Data pre-processing and analyses was performed using the Statistical Parametric Mapping software 8 (SPM8; http://www.fil.ion.ucl.ac.uk/spm/) implemented in Matlab 7.5 (Mathworks, Natick, Massachusetts, US). Within each subject all volumes were aligned to the first volume, and the images were then spatially normalized to a standard EPI template based upon the Montreal Neurological Institute (MNI) reference brain and resampled at a voxel size of 3 x 3 x 3 mm. The images were smoothed using an 8 mm full-width at half maximum Gaussian isotropic kernel.

#### GLM analysis

A general linear model was constructed by convolving stick functions with a canonical hemodynamic response function. The model consisted of ten regressors: time onsets for *Cue for aversive stimulus* and *Cue for neutral stimulus* with or without outcome, in addition to six movement parameters. The trials with an outcome were not used in any contrasts because of risk of contaminating the BOLD signal related to delivery of sound. Possible slow signal drift was removed with a high-pass filter with a 128 s cut-off. The contrast *Cue for aversive stimulus without outcome* vs. *Cue for neutral stimulus without outcome* was moved to second-level random-effects one-sample t-test analyses.

The *a priori* regions of interest (ROIs) were the ventral striatum (VS), the amygdala, the anterior cingulate cortex (ACC) and the anterior insula. Bilateral masks were created using the Automated anatomical labeling (aal) atlas in the SPM Wake Forest University (WFU) PickAtlas toolbox (http://fmri.wfubmc.edu/software/PickAtlas, version 2.5 [23,24]). Only the anterior part of the aal insula mask was used (y > 10). Since an appropriate mask for the VS was not available in the PickAtlas, a mask from the BrainMap database was used [25,26]. Small volume corrections were performed within each bilateral mask and the threshold levels were adapted to the four ROIs to account for multiple comparisons by Bonferroni corrections.

#### Psychophysiological interaction (PPI) analysis

During a psychophysiological interaction (PPI) analysis possible interactions between regression slopes of different brain areas can be significance tested as a measure of regional connectivity. Two separate PPI analyses were performed. The left VS served as the seed region for one of the PPI analyses. The left side was chosen because this side yielded stronger activations in the initial GLM analysis. Similarly, the right side amygdala served as seed region for the other PPI analysis. For each subject mean corrected activity (first eigenvariates) from 6 mm radius spheres centered on individual peak activation voxels within the seeds were extracted. The extracted haemodynamic time series were then deconvolved and the resulting neuronal time series (physiological variable) were combined with the onset times for *Cue for aversive stimulus* and *Cue for neutral stimulus* (psychological variable) to derive the interaction term (source signal * experimental treatment). To test for differences in regression slopes between the two experimental conditions a GLM was generated with this interaction term as the explanatory variable. The resulting individual t-contrast images were entered into a random effects group analysis and tested for statistical significance. To test the hypotheses that the functional connectivity between the VS and the OFC, and the amygdala and the OFC would be stronger during aversive trials than neutral trials, small volume correction within the left OFC was employed. The OFC mask included the superior, middle and medial parts of the OFC as defined in the aal atlas in the WFU PickAtlas toolbox (http://fmri.wfubmc.edu/software/PickAtlas, version 2.5 [23]).

### *Behavior analysis*

The behavioral data were analyzed using Statistical Package for the Social Sciences (SPSS, Version 14, SPSS Inc., Chicago, Illinois, USA).

The data material will be made available upon request.

## Results

### *Behavior analysis*

Analysis of the behavioral data showed a significant difference between response times on targets in aversive and in non-aversive trials, with responses being faster during aversive conditions (213 ± 20 ms vs. 240 ± 36 ms, t = 4.04; p < 0.001). The outcome schedule was adjusted during the task based on individual response times so that the total number of trials with outcomes would be approximately 20% in both conditions. The subjects were more successful avoiding the sounds in aversive trials than neutral trials (82.7 ± 3.1% aversive trials and 80.2 ± 4.0% neutral trials, t = 3.30; p < 0.01).

### *Image analysis*

**Figure 1:**
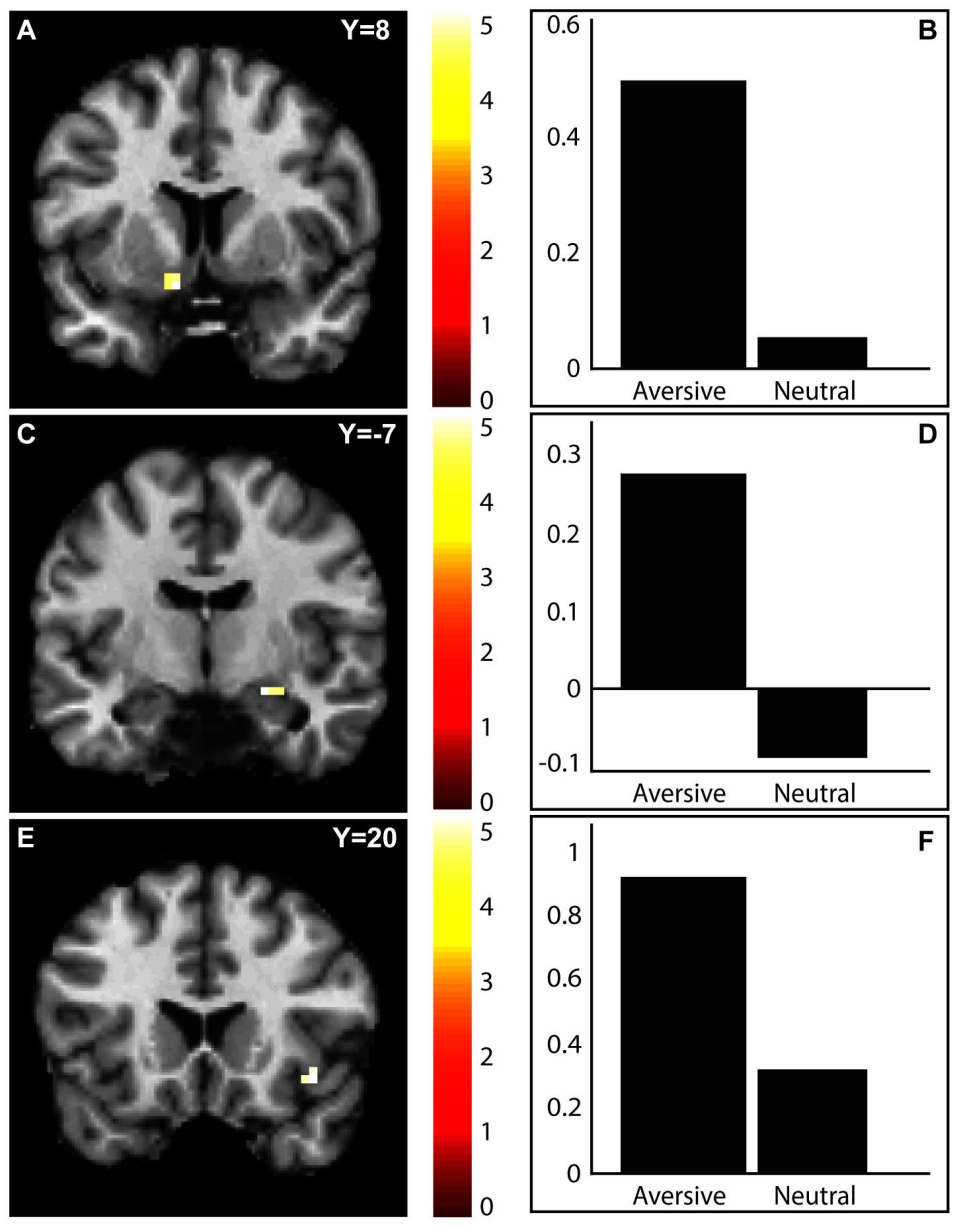
fMRI BOLD activation for the contrast Cue for aversive stimulus > Cue for neutral stimulus. Statistical parametric maps (SPM) showing significant activations in the ventral striatum (A), the amygdala (C) and the insula (E). Colors indicate t-values of activated voxels and are coded in the bars on the right. Peak voxel beta values are shown for the two conditions in the ventral striatum (B), the amygdala (D) and the insular cortex (F). Family wise error corrected within bilateral ROIs at threshold *p* < 0.05, and Bonferroni corrected to control for tests of multiple ROIs.

*A priori* ROI analyses of the VS, the amygdala, the ACC and the insula were performed. Bonferroni corrected significant activations were found in the bilateral VS, the right amygdala and the right anterior insula during the *Cue for aversive stimulus* > *Cue for neutral stimulus* contrast (Figure 1 and Table 1). Whole brain analysis yielded no significant activations when correcting for multiple comparisons.

**Table 1:** List of peak voxels of significant clusters in the region of interest analyses.

Region	Hemisphere	Coordinates *x, y, z*			Z	*P*_FWE_
Ventral striatum	Left	-9	8	-14	3.80	<0.005
	Right	18	2	-8	3.68	<0.01
Amygdala	Left	-27	2	-17	3.31	N.S.
	Right	21	-7	-14	3.65	<0.005
Anterior insula	Left	-33	20	-11	3.23	N.S.
	Right	42	20	-11	3.87	<0.01

Bonferroni correction is applied to control for multiple comparisons.

**Figure 2:**
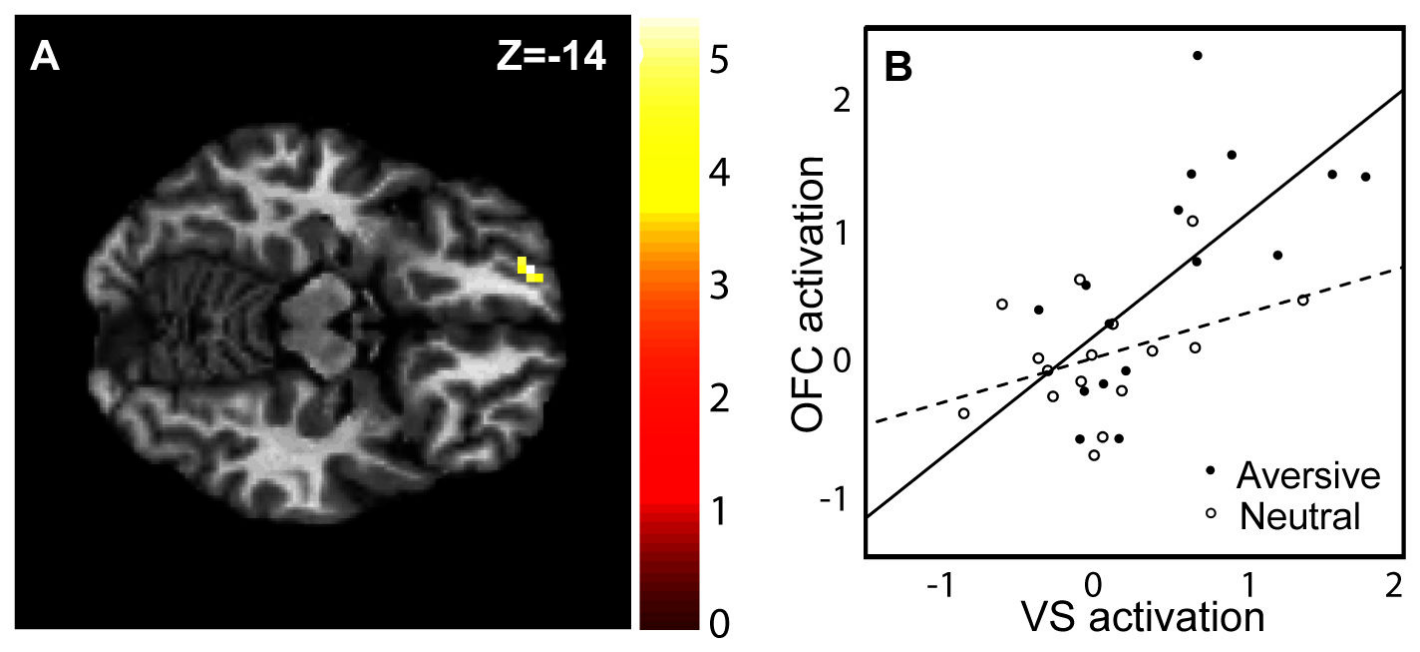
Results of the psychophysiological interaction (PPI) analysis. (A) Statistical parametric map (SPM) showing the OFC voxel cluster that showed differences in interaction strength between the two conditions aversive and neutral. Colors indicate t-values of activated voxels and are coded in the bar on the right. Family wise error corrected within ROI at threshold *p* < 0.05 (B) The interaction found between the ventral striatum (VS) and the orbitofrontal cortex (OFC) is plotted with regression lines. The solid line represents aversive condition and the dashed line represents neutral condition. Beta values of the VS are indicated on the x-axis and beta values of the OFC are indicated on the y axis.

The PPI analysis showed that the connectivity between the left VS and the left OFC significantly increased when subjects viewed cues for aversive sound as compared to neutral sound (Figure 2). The peak voxel of the orbitofrontal cluster (k = 7) was found at x = -18, y = 56, z = -14, *p*_FWE(svc)_ = 0.01. These voxels were tested and found to be more active during presentation of aversive than neutral cues *p*_FWE_ < 0,05. The interaction between the amygdala and the OFC did not show a significant increase for the same contrast (*p*_FWE_ = 0.12).

## Discussion

The VS, insula and amygdala showed activations when we targeted avoidance anticipation behavior with an fMRI task based on avoidance. This replicates previous findings obtained with a similar task but different stimulus modality [5]. Further, a stronger covariation between activations in the VS and the OFC was present during salient cues for aversive events as compared to cues for non-aversive events, suggesting that the OFC might be involved in coding of incentive salience.

There was greater connectivity between the VS and the OFC when viewing cues associated with an aversive event. Both the VS and the OFC are targeted by dopamine and play important roles in reward and motivation [27]. The function the OFC serves in motivation, however, is still debated. Several functions have been proposed, such as converting various stimuli of different modalities into a common currency in order to compare stimuli values [28], and inhibiting undesired behavior [22]. The mesocorticolimbic system has traditionally been conceptualized as a reward system and much of the literature has focused on appetitive conditioning. However, it has been suggested that this network plays an important role in processing of aversive information [5–7]. Similarly, it is generally accepted that the OFC represents both rewarding and aversive stimuli [29], but its role in motivation is still not resolved and few studies yet have investigated this issue.

The connectivity analysis using VS as a seed region yielded a cluster in the left superior orbital gyrus. This finding is consistent with a suggested role for the OFC in motivation related to VS function. Since neurons in the OFC represent intensity of a conditioned stimulus in a graded fashion [30], it has been suggested that the main responsibility of this region may be to integrate information of outcomes and track value of the subject’s state in ongoing representations of motivational value. In line with this, one can speculate that the OFC activity found in the VS–OFC connectivity analysis in the present study may represent the association between a cue and the motivational significance of the outcome that the cue predicts [30]. An increase in connectivity between the amygdala and the OFC during presentation of salient cues was hypothesized, but no significant increase was found. The OFC and the amygdala are reciprocally connected and are thought to be involved in similar processes [22]. Both regions have been found to be more active during emotional association learning compared to after an association is learned. As the paradigm in the present study did not involve learning this might explain the lack of increase in connectivity.

The VS has earlier been linked to experiencing, predicting and anticipating both appetitive and aversive events [7,31]. In the present study greater activations were shown in the VS during salient cues that predicted an avoidable aversive event. This is consistent with a function in active avoidance that has previously been suggested for the ventral striatum. Early evidence from animal studies showed that the ventral midbrain is important for the initiation of goal-directed behavior, bridging motivation and action [32]. Building on this notion of the ventral midbrain as an initiator of action, several studies have implicated the VS in motivation and goal-directed behavior [33,34]. While much of this work has focused on the VS’s role in reward, some studies have used aversive stimuli. For example, in an fMRI study by Delgado et al. [7], trials where subjects were told that they could avoid aversive events, but had not yet learned how, were compared with trials where they could not avoid the aversive outcomes. Stronger activations in the VS were found in the trials where subjects thought they could avoid the aversive outcome. The activations found in the present study are in line with these findings. However, Delgado et al. [7] targeted avoidance learning and found that once the subjects learned how the aversive outcomes could be avoided the effect was no longer present. Interestingly, as the subjects in the present study were informed about which cue predicted the aversive sound and how to act to avoid it, it is less likely that there was any learning going on, and one can speculate that the striatal activation in the present study might rather represent incentive salience and preparation to act. However, while the present study employed a task based on active avoidance, previous work has also shown striatal activation during mere expectation of aversive stimulus. Jensen et al (2003), found ventral striatal activity during both active avoidance and passive expectation of aversive events, but these were not directly compared. One way to explore how these conditions relate to one another could be to implement passive trials in the present paradigm in order to directly compare active avoidance with passive non-reinforced trials.

In the present study the amygdala showed stronger activation in the negative, and presumably more salient, trials. It has been shown that the amygdala responds in a graded fashion to the importance of a stimulus [35] and recent reports indicate that the amygdala plays a role in detecting stimuli associated with personal relevance [18,19,36]. In line with this work, the present finding of stronger activation during salient trials may suggest that the amygdala encodes some kind of relevance related feature. The amygdala has generally been accepted as a key structure in fear processing and is known to be activated during fear conditioning as well as during expression of learned fear [17]. More recently the amygdala has also been linked with other emotional contents and is now suggested to serve a more general role in relevance detection [19,21]. Based on these findings and the abundant interconnections between amygdala and different parts of the sensory and perceptual systems that enables it to influence attention and contribute to preparation for action [37], amygdala activations were expected when contrasting presentation of salient cues and corresponding neutral cues. Using a similar paradigm Jensen et al. [5] reported no amygdala activations, but this might have been caused by signal dropout in this region. The amygdala activation reported in the present study links the newly established role for amygdala as a relevance detector with incentive salience. One can speculate that the amygdala is involved in the earlier stage of the process where it might create information about personal relevance of events and convey this to the salience-processing system.

Possible activations in the insula and ACC in the current study is of interest because these regions have previously been linked to the anticipation of negative outcomes [38], and are found to be important in fear conditioning [39]. Both the insula and ACC have previously been reported to activate during experience of aversion [38,40], expression of learned fear [41] and anticipation of aversive events [5,16,42], and are thought to be critical in avoidance behavior. In the present study the right anterior insula yielded stronger activation during aversive than neutral trials, and this is in line with previous work [13]. Recent findings have suggested that the function of the anterior insula in regard to avoidance anticipation may be to tag salient events for further processing and to initiate appropriate control signaling [13]. Based on this, one may speculate that the anterior insula activation in the present study represent cue tagging and thus indicates a role for this region in salience processing. The insula has previously been found to be involved in processing of diverse aversive emotions [43,44] and to be central in risk processing [45]. The insular cortex is reciprocally connected with the amygdala [46], and it has been suggested that the insula may relay fear information from the amygdala to the cerebral cortex, and also relay cortical representations of the anticipated event the opposite way [40]. The VS also receives projections from the insula [47], and thus gets processed fear-related information. This information would be relevant for the motivational control of goal-directed behavior that the VS is thought to be involved in [33]. Anticipation of outcome has repeatedly been shown to activate the anterior insula, and anticipation of negative outcome is reported to activate the anterior insula more than positive outcome [14]. The present finding is in line with these reports as the anterior insula activated during anticipation of negative outcome, but the present study did not allow for comparisons between anticipation/outcome or negative/positive outcome. The lack of significant activations in the ACC was somewhat unexpected as this region has been reported to show activations during similar tasks and to be associated with various functions that could be linked to motivational control of behavior.

In conclusion, the current findings of a stronger connectivity between the OFC and the VS during avoidance preparation of aversive events support a role for the OFC in incentive salience. Further, the replication of previous findings from studies using a similar task but with other stimuli modalities corroborate the robustness of the avoidance type task employed.
